# *N*-[(9-Ethyl-9*H*-carbazol-3-yl)methyl­idene]-3,4-dimethyl­isoxazol-5-amine

**DOI:** 10.1107/S1600536810027686

**Published:** 2010-07-17

**Authors:** Abdullah M. Asiri, Salman A. Khan, Kong Wai Tan, Seik Weng Ng

**Affiliations:** a Chemistry Department, Faculty of Science, King Abdul Aziz University, PO Box 80203, Jeddah 21589, Saudi Arabia; b Department of Chemistry, University of Malaya, 50603 Kuala Lumpur, Malaysia

## Abstract

The azomethine double bond in the title Schiff base, C_20_H_19_N_3_O, has an *E* configuration. The 13-membered carbazolyl fused ring system [r.m.s. deviation = 0.023 (9) Å] is nearly coplanar with the five-membered pyrazole ring [r.m.s. deviation = 0.003 (4) Å]; the dihedral angle between the two systems is 10.8 (2)°. The crystal studied was a non-merohedral twin having a 35% minor component.

## Related literature

For the synthesis and spectroscopic characterization of the title compound, see: Asiri *et al.* (2010 ▶). For the treatment of non-merohedral twins, see: Spek (2009 ▶).
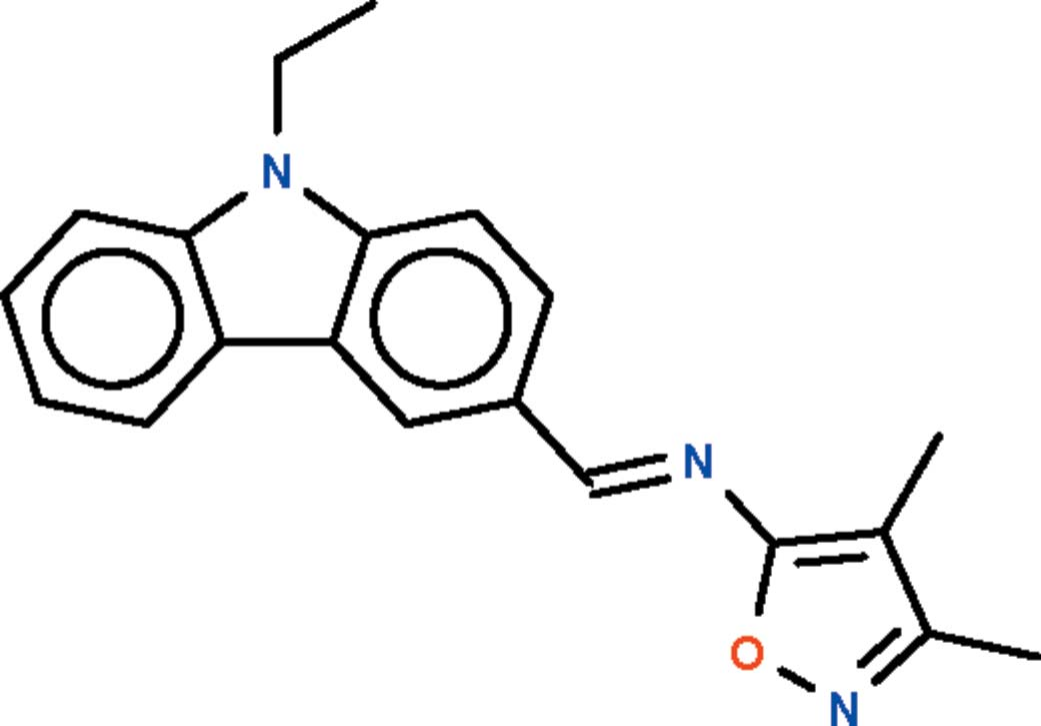


## Experimental

### 

#### Crystal data

C_20_H_19_N_3_O
*M* *_r_* = 317.38
Monoclinic, 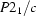

*a* = 8.0575 (9) Å
*b* = 13.4483 (15) Å
*c* = 14.8488 (16) Å
β = 94.049 (2)°
*V* = 1605.0 (3) Å^3^
*Z* = 4
Mo *K*α radiation
μ = 0.08 mm^−1^
*T* = 100 K
0.20 × 0.15 × 0.05 mm

#### Data collection

Bruker SMART APEX diffractometer
11993 measured reflections
2811 independent reflections
2286 reflections with *I* > 2σ(*I*)
*R*_int_ = 0.057

#### Refinement

*R*[*F* ^2^ > 2σ(*F* ^2^)] = 0.089
*wR*(*F* ^2^) = 0.262
*S* = 1.11
2811 reflections
221 parameters
H-atom parameters constrained
Δρ_max_ = 0.55 e Å^−3^
Δρ_min_ = −0.54 e Å^−3^

### 

Data collection: *APEX2* (Bruker, 2009 ▶); cell refinement: *SAINT* (Bruker, 2009 ▶); data reduction: *SAINT*; program(s) used to solve structure: *SHELXS97* (Sheldrick, 2008 ▶); program(s) used to refine structure: *SHELXL97* (Sheldrick, 2008 ▶); molecular graphics: *X-SEED* (Barbour, 2001 ▶); software used to prepare material for publication: *publCIF* (Westrip, 2010 ▶).

## Supplementary Material

Crystal structure: contains datablocks global, I. DOI: 10.1107/S1600536810027686/rk2213sup1.cif

Structure factors: contains datablocks I. DOI: 10.1107/S1600536810027686/rk2213Isup2.hkl

Additional supplementary materials:  crystallographic information; 3D view; checkCIF report

## Figures and Tables

**Fig. 1.**
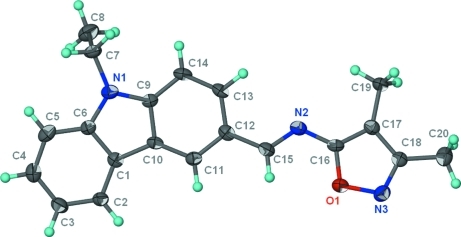
The molecular structure of title compound with the atom numbering scheme. Displacement ellipsoids are drawn at 70% probability level. Hydrogen atoms are drawn as a small spheres of arbitrary radius.

## supplementary crystallographic information

### Comment

We have recently reported the synthesis and characterization of this Schiff base (Asiri *et al.*, 2010). The azomethine double-bond of the Schiff base (Scheme, Fig. 1) has an *E*-configuration. The 13-membered carbazolyl fused-ring (r.m.s. deviation 0.023 (9)Å) is nearly coplanar with 5-membered pyrazolyl ring (r.m.s. deviation 0.003 (4)Å) (dihedral angle between the two systems 10.8 (2)°).

### Experimental

Crystals of the compound were synthesized as reported (Asiri *et al.*, 2010).

### Refinement

Carbon-bound H-atoms were placed in calculated positions [C—H 0.95 to 0.98Å with *U*_iso_(H) 1.2 to 1.5*U*_eq_(C)] and were included in the refinement in the riding model approximation.

The crystal studied is a non-merohedral twin; *PLATON* (Spek, 2009) gave the twin law as (-1 0 0, 0 -1 0, 0.260 0 -1). The de-twinning option lowered the *R* index from 17% to a more acceptable level. However, the twinning affected the refinement so that the thermal ellipsoids, particularly those of the heterocyclic ring, were somewhat large.

### Crystal data

**Table:**

C_20_H_19_N_3_O	*F*(000) = 672
*M**_r_* = 317.38	*D*_x_ = 1.313 Mg m^−^^3^
Monoclinic, *P*2_1_/*c*	Mo *K*α radiation, λ = 0.71073 Å
Hall symbol: -P 2ybc	Cell parameters from 3958 reflections
*a* = 8.0575 (9) Å	θ = 2.5–27.4°
*b* = 13.4483 (15) Å	µ = 0.08 mm^−^^1^
*c* = 14.8488 (16) Å	*T* = 100 K
β = 94.049 (2)°	Prism, yellow
*V* = 1605.0 (3) Å^3^	0.20 × 0.15 × 0.05 mm
*Z* = 4	

### Data collection

**Table:**

Bruker SMART APEX diffractometer	2286 reflections with *I* > 2σ(*I*)
Radiation source: fine-focus sealed tube	*R*_int_ = 0.057
graphite	θ_max_ = 25.0°, θ_min_ = 2.1°
ω scans	*h* = −9→8
11993 measured reflections	*k* = −15→15
2811 independent reflections	*l* = −17→17

### Refinement

**Table:**

Refinement on *F*^2^	Primary atom site location: structure-invariant direct methods
Least-squares matrix: full	Secondary atom site location: difference Fourier map
*R*[*F*^2^ > 2σ(*F*^2^)] = 0.089	Hydrogen site location: inferred from neighbouring sites
*wR*(*F*^2^) = 0.262	H-atom parameters constrained
*S* = 1.11	*w* = 1/[σ^2^(*F*_o_^2^) + (0.1086*P*)^2^ + 4.6936*P*] where *P* = (*F*_o_^2^ + 2*F*_c_^2^)/3
2811 reflections	(Δ/σ)_max_ = 0.001
221 parameters	Δρ_max_ = 0.55 e Å^−^^3^
0 restraints	Δρ_min_ = −0.54 e Å^−^^3^

### Special details

**Table:**

Geometry. All s.u.'s (except the s.u. in the dihedral angle between two l.s. planes) are estimated using the full covariance matrix. The cell s.u.'s are taken into account individually in the estimation of s.u.'s in distances, angles and torsion angles; correlations between s.u.'s in cell parameters are only used when they are defined by crystal symmetry. An approximate (isotropic) treatment of cell s.u.'s is used for estimating s.u.'s involving l.s. planes.
Refinement. Refinement of *F*^2^ against ALL reflections. The weighted *R*-factor wR and goodness of fit *S* are based on *F*^2^, conventional *R*-factors *R* are based on *F*, with *F* set to zero for negative *F*^2^. The threshold expression of *F*^2^ > σ(*F*^2^) is used only for calculating *R*-factors(gt) etc. and is not relevant to the choice of reflections for refinement. *R*-factors based on *F*^2^ are statistically about twice as large as those based on *F*, and *R*-factors based on ALL data will be even larger.

### Fractional atomic coordinates and isotropic or equivalent isotropic displacement parameters (Å^2^)

**Table:**

	*x*	*y*	*z*	*U*_iso_*/*U*_eq_	
O1	0.2910 (4)	0.3151 (2)	0.1571 (2)	0.0238 (8)	
N1	1.1980 (5)	0.1389 (3)	0.4076 (3)	0.0219 (9)	
N2	0.4716 (5)	0.1815 (3)	0.2053 (3)	0.0216 (9)	
N3	0.1279 (5)	0.3252 (3)	0.1158 (3)	0.0253 (9)	
C1	1.1728 (6)	0.3018 (3)	0.3684 (3)	0.0187 (10)	
C2	1.2291 (6)	0.3999 (3)	0.3616 (3)	0.0215 (10)	
H2	1.1602	0.4489	0.3320	0.026*	
C3	1.3860 (6)	0.4244 (3)	0.3984 (3)	0.0257 (11)	
H3	1.4247	0.4909	0.3947	0.031*	
C4	1.4879 (6)	0.3522 (4)	0.4411 (3)	0.0257 (11)	
H4	1.5947	0.3708	0.4667	0.031*	
C5	1.4375 (6)	0.2540 (4)	0.4472 (3)	0.0248 (11)	
H5	1.5084	0.2049	0.4753	0.030*	
C6	1.2794 (6)	0.2302 (3)	0.4105 (3)	0.0185 (10)	
C7	1.2634 (6)	0.0460 (3)	0.4467 (3)	0.0239 (11)	
H7A	1.1712	0.0081	0.4710	0.029*	
H7B	1.3453	0.0614	0.4976	0.029*	
C8	1.3456 (7)	−0.0178 (4)	0.3792 (4)	0.0336 (13)	
H8A	1.3818	−0.0805	0.4078	0.050*	
H8B	1.4423	0.0174	0.3583	0.050*	
H8C	1.2662	−0.0315	0.3277	0.050*	
C9	1.0436 (6)	0.1505 (3)	0.3641 (3)	0.0175 (10)	
C10	1.0204 (6)	0.2514 (3)	0.3393 (3)	0.0180 (10)	
C11	0.8678 (6)	0.2813 (3)	0.2978 (3)	0.0193 (10)	
H11	0.8495	0.3491	0.2821	0.023*	
C12	0.7420 (6)	0.2112 (3)	0.2796 (3)	0.0212 (10)	
C13	0.7718 (6)	0.1102 (3)	0.3024 (3)	0.0212 (10)	
H13	0.6869	0.0627	0.2875	0.025*	
C14	0.9185 (6)	0.0784 (3)	0.3453 (3)	0.0228 (11)	
H14	0.9354	0.0107	0.3616	0.027*	
C15	0.5842 (6)	0.2432 (3)	0.2365 (3)	0.0205 (10)	
H15	0.5625	0.3124	0.2311	0.025*	
C16	0.3229 (6)	0.2162 (3)	0.1664 (3)	0.0186 (10)	
C17	0.1911 (6)	0.1619 (3)	0.1324 (3)	0.0207 (10)	
C18	0.0728 (6)	0.2352 (3)	0.1022 (3)	0.0197 (10)	
C19	0.1746 (6)	0.0520 (3)	0.1276 (3)	0.0253 (11)	
H19A	0.2802	0.0211	0.1493	0.038*	
H19B	0.1463	0.0320	0.0650	0.038*	
H19C	0.0866	0.0304	0.1655	0.038*	
C20	−0.0982 (6)	0.2179 (4)	0.0589 (3)	0.0280 (11)	
H20A	−0.1665	0.1847	0.1020	0.042*	
H20B	−0.0911	0.1758	0.0054	0.042*	
H20C	−0.1488	0.2818	0.0410	0.042*	

### Atomic displacement parameters (Å^2^)

**Table:**

	*U*^11^	*U*^22^	*U*^33^	*U*^12^	*U*^13^	*U*^23^
O1	0.0252 (18)	0.0164 (16)	0.0296 (18)	−0.0002 (14)	0.0004 (14)	0.0000 (13)
N1	0.026 (2)	0.0186 (19)	0.021 (2)	0.0010 (17)	0.0022 (17)	0.0028 (15)
N2	0.023 (2)	0.0176 (19)	0.024 (2)	−0.0013 (17)	0.0046 (17)	0.0003 (15)
N3	0.024 (2)	0.024 (2)	0.028 (2)	0.0035 (18)	0.0010 (18)	0.0026 (17)
C1	0.022 (2)	0.019 (2)	0.015 (2)	−0.0006 (19)	0.0041 (18)	−0.0030 (17)
C2	0.021 (2)	0.016 (2)	0.027 (2)	0.0013 (19)	0.002 (2)	−0.0006 (18)
C3	0.024 (3)	0.019 (2)	0.034 (3)	−0.003 (2)	0.004 (2)	−0.003 (2)
C4	0.021 (2)	0.031 (3)	0.024 (2)	−0.001 (2)	0.001 (2)	−0.004 (2)
C5	0.029 (3)	0.027 (2)	0.019 (2)	0.009 (2)	0.001 (2)	0.0007 (19)
C6	0.021 (2)	0.018 (2)	0.017 (2)	0.0017 (19)	0.0020 (18)	−0.0010 (17)
C7	0.031 (3)	0.016 (2)	0.025 (2)	0.003 (2)	−0.002 (2)	0.0036 (18)
C8	0.037 (3)	0.027 (3)	0.037 (3)	0.009 (2)	0.000 (2)	0.002 (2)
C9	0.021 (2)	0.016 (2)	0.015 (2)	0.0030 (18)	0.0046 (19)	0.0001 (17)
C10	0.024 (3)	0.014 (2)	0.016 (2)	−0.0009 (18)	0.0040 (19)	−0.0010 (16)
C11	0.023 (3)	0.017 (2)	0.018 (2)	0.0014 (19)	0.0032 (19)	−0.0007 (17)
C12	0.024 (3)	0.021 (2)	0.019 (2)	−0.003 (2)	0.0043 (19)	−0.0026 (18)
C13	0.025 (3)	0.017 (2)	0.022 (2)	−0.0061 (19)	0.005 (2)	−0.0020 (18)
C14	0.030 (3)	0.013 (2)	0.025 (2)	−0.001 (2)	0.005 (2)	0.0007 (18)
C15	0.021 (2)	0.017 (2)	0.024 (2)	−0.0023 (19)	0.005 (2)	−0.0019 (18)
C16	0.023 (2)	0.014 (2)	0.019 (2)	0.0001 (18)	0.0054 (19)	0.0019 (17)
C17	0.021 (2)	0.019 (2)	0.022 (2)	−0.0016 (19)	0.0039 (19)	−0.0006 (18)
C18	0.021 (2)	0.020 (2)	0.018 (2)	−0.0012 (19)	0.0040 (19)	0.0003 (18)
C19	0.024 (3)	0.016 (2)	0.036 (3)	−0.002 (2)	−0.003 (2)	0.0011 (19)
C20	0.025 (3)	0.033 (3)	0.026 (3)	0.000 (2)	0.000 (2)	0.001 (2)

### Geometric parameters (Å, °)

**Table:**

O1—C16	1.360 (5)	C8—H8B	0.9800
O1—N3	1.417 (5)	C8—H8C	0.9800
N1—C9	1.369 (6)	C9—C14	1.412 (6)
N1—C6	1.391 (6)	C9—C10	1.416 (6)
N1—C7	1.461 (6)	C10—C11	1.395 (6)
N2—C15	1.291 (6)	C11—C12	1.397 (6)
N2—C16	1.375 (6)	C11—H11	0.9500
N3—C18	1.300 (6)	C12—C13	1.416 (6)
C1—C2	1.400 (6)	C12—C15	1.448 (7)
C1—C6	1.407 (6)	C13—C14	1.371 (7)
C1—C10	1.443 (6)	C13—H13	0.9500
C2—C3	1.381 (7)	C14—H14	0.9500
C2—H2	0.9500	C15—H15	0.9500
C3—C4	1.395 (7)	C16—C17	1.357 (6)
C3—H3	0.9500	C17—C18	1.422 (6)
C4—C5	1.386 (7)	C17—C19	1.485 (6)
C4—H4	0.9500	C18—C20	1.496 (7)
C5—C6	1.387 (7)	C19—H19A	0.9800
C5—H5	0.9500	C19—H19B	0.9800
C7—C8	1.507 (7)	C19—H19C	0.9800
C7—H7A	0.9900	C20—H20A	0.9800
C7—H7B	0.9900	C20—H20B	0.9800
C8—H8A	0.9800	C20—H20C	0.9800
			
C16—O1—N3	107.5 (3)	C11—C10—C9	119.2 (4)
C9—N1—C6	109.0 (4)	C11—C10—C1	134.6 (4)
C9—N1—C7	125.1 (4)	C9—C10—C1	106.2 (4)
C6—N1—C7	125.9 (4)	C10—C11—C12	119.7 (4)
C15—N2—C16	120.1 (4)	C10—C11—H11	120.1
C18—N3—O1	105.9 (4)	C12—C11—H11	120.1
C2—C1—C6	119.1 (4)	C11—C12—C13	119.5 (4)
C2—C1—C10	134.0 (4)	C11—C12—C15	119.2 (4)
C6—C1—C10	107.0 (4)	C13—C12—C15	121.3 (4)
C3—C2—C1	119.2 (4)	C14—C13—C12	122.4 (4)
C3—C2—H2	120.4	C14—C13—H13	118.8
C1—C2—H2	120.4	C12—C13—H13	118.8
C2—C3—C4	120.4 (4)	C13—C14—C9	117.3 (4)
C2—C3—H3	119.8	C13—C14—H14	121.4
C4—C3—H3	119.8	C9—C14—H14	121.4
C5—C4—C3	121.8 (5)	N2—C15—C12	122.7 (4)
C5—C4—H4	119.1	N2—C15—H15	118.6
C3—C4—H4	119.1	C12—C15—H15	118.6
C4—C5—C6	117.3 (4)	C17—C16—O1	110.6 (4)
C4—C5—H5	121.3	C17—C16—N2	127.6 (4)
C6—C5—H5	121.3	O1—C16—N2	121.9 (4)
C5—C6—N1	129.3 (4)	C16—C17—C18	103.5 (4)
C5—C6—C1	122.1 (4)	C16—C17—C19	128.2 (4)
N1—C6—C1	108.6 (4)	C18—C17—C19	128.3 (4)
N1—C7—C8	112.7 (4)	N3—C18—C17	112.5 (4)
N1—C7—H7A	109.1	N3—C18—C20	120.4 (4)
C8—C7—H7A	109.1	C17—C18—C20	127.2 (4)
N1—C7—H7B	109.1	C17—C19—H19A	109.5
C8—C7—H7B	109.1	C17—C19—H19B	109.5
H7A—C7—H7B	107.8	H19A—C19—H19B	109.5
C7—C8—H8A	109.5	C17—C19—H19C	109.5
C7—C8—H8B	109.5	H19A—C19—H19C	109.5
H8A—C8—H8B	109.5	H19B—C19—H19C	109.5
C7—C8—H8C	109.5	C18—C20—H20A	109.5
H8A—C8—H8C	109.5	C18—C20—H20B	109.5
H8B—C8—H8C	109.5	H20A—C20—H20B	109.5
N1—C9—C14	128.9 (4)	C18—C20—H20C	109.5
N1—C9—C10	109.3 (4)	H20A—C20—H20C	109.5
C14—C9—C10	121.8 (4)	H20B—C20—H20C	109.5
			
C16—O1—N3—C18	−0.6 (5)	C2—C1—C10—C9	178.0 (5)
C6—C1—C2—C3	−1.7 (6)	C6—C1—C10—C9	−1.0 (5)
C10—C1—C2—C3	179.5 (5)	C9—C10—C11—C12	−1.5 (6)
C1—C2—C3—C4	0.6 (7)	C1—C10—C11—C12	−179.1 (5)
C2—C3—C4—C5	0.9 (7)	C10—C11—C12—C13	−0.8 (6)
C3—C4—C5—C6	−1.2 (7)	C10—C11—C12—C15	180.0 (4)
C4—C5—C6—N1	−179.6 (4)	C11—C12—C13—C14	2.5 (7)
C4—C5—C6—C1	0.1 (7)	C15—C12—C13—C14	−178.3 (4)
C9—N1—C6—C5	−179.6 (4)	C12—C13—C14—C9	−1.8 (7)
C7—N1—C6—C5	2.0 (7)	N1—C9—C14—C13	178.3 (4)
C9—N1—C6—C1	0.6 (5)	C10—C9—C14—C13	−0.6 (6)
C7—N1—C6—C1	−177.8 (4)	C16—N2—C15—C12	178.9 (4)
C2—C1—C6—C5	1.3 (6)	C11—C12—C15—N2	169.3 (4)
C10—C1—C6—C5	−179.6 (4)	C13—C12—C15—N2	−9.9 (7)
C2—C1—C6—N1	−178.9 (4)	N3—O1—C16—C17	0.9 (5)
C10—C1—C6—N1	0.2 (5)	N3—O1—C16—N2	−179.4 (4)
C9—N1—C7—C8	86.7 (6)	C15—N2—C16—C17	−178.4 (4)
C6—N1—C7—C8	−95.2 (5)	C15—N2—C16—O1	2.0 (6)
C6—N1—C9—C14	179.8 (4)	O1—C16—C17—C18	−0.9 (5)
C7—N1—C9—C14	−1.9 (7)	N2—C16—C17—C18	179.5 (4)
C6—N1—C9—C10	−1.2 (5)	O1—C16—C17—C19	178.9 (4)
C7—N1—C9—C10	177.1 (4)	N2—C16—C17—C19	−0.7 (8)
N1—C9—C10—C11	−176.9 (4)	O1—N3—C18—C17	0.1 (5)
C14—C9—C10—C11	2.2 (6)	O1—N3—C18—C20	−179.9 (4)
N1—C9—C10—C1	1.4 (5)	C16—C17—C18—N3	0.5 (5)
C14—C9—C10—C1	−179.6 (4)	C19—C17—C18—N3	−179.3 (5)
C2—C1—C10—C11	−4.2 (9)	C16—C17—C18—C20	−179.5 (4)
C6—C1—C10—C11	176.8 (5)	C19—C17—C18—C20	0.7 (8)
